# Research collaboration with low resource countries: overcoming the challenges

**DOI:** 10.1186/1750-9378-6-S2-S3

**Published:** 2011-09-23

**Authors:** Titilola O Akinremi

**Affiliations:** 1 Dept of Pathology, Federal Medical Center, Abeokuta, Nigeria

## Abstract

The gap in research capacity between low and high resource countries and the effect on the global trend of health research cannot be ignored any more. Therefore the need for research collaboration between the two groups cannot be overemphasized. The discrepancy of economies makes for differences in research capacity, psychosocial needs, ethical considerations, focus and challenges. For such collaboration to work out, every collaborator needs to be aware of these challenges and work with them. Collaborations with low resource countries will require transparency, mutual respect and respect for social norms. Collaborations should no longer be that of taker or master, but of cooperation. The dearth of adequate research manpower and well equipped laboratories in low resource countries calls for a deliberate effort by strong research centers to go a step further in collaboration and adopt a centre or laboratory.

## Introduction

Research collaboration simply put, is the working together of researchers to achieve a ***common goal*** of producing scientific knowledge [1]. It includes: partnering with colleagues to engage in knowledge and innovation production, formal and informal engagement in consultations, advice, visits, conferences or exchange programs, sharing facilities and knowledge banks/data, virtual networking and training.

Lots of benefits could be derived from research collaboration, including tapping into global knowledge in areas of interest and more, as well as the development of networks for joint initiatives and knowledge exchange [2]. It may also lead to personal professional development and open doors to greater opportunities for research funding, facility improvement, and scholarships [3].

Research partnership involves deciding on the objectives of the research together, building up mutual trust among partners and sharing information freely among partners [4]. The principles of research partnership also include developing networks, sharing responsibility and ensuring transparency among partners. Research collaborators share responsibility in monitoring and evaluating, disseminating results, applying results, equitable sharing of profits, capacity building, as well as building on achievements [5]. Research collaborators include people who jointly prepared the original proposal, work on the project, and/or are responsible for one or more of the main part of the research. They become the co-authors [6].

## Objective

The aim of this study is to attempt to identify the challenges of research collaborations with low resource countries and proffer some solutions to such.

## Methods

This is a review of the literature about the practice and challenges of international research collaborations involving low resource countries particularly as it applies to health research. The National Library of Medicine search engine, Pubmed [7] and Google [8] were used to identify relevant literature with the terms “research collaboration”, and “low resource” or“ low economy ”and “health research”.

## Why collaborate?

The global trend of funding requirements has moved towards collaborative research in recent times and researchers are encouraged to join forces inter-departmentally, inter-institutionally, and even internationally [1]. Outcomes of such research are better respected than single authored ones, so that researcher’s need for recognition also drives collaboration. The growing complexity of expensive research equipments also calls for collaborative efforts so that these facilities can be shared with greater cost effectiveness. In the same way, scientific manpower could be shared without the need for each research center to acquire all areas of manpower. While research capacity is measured by skills and competencies, scientific activities, outcomes, and impacts on policies and programs, the impact of research collaboration is measured by co-authorship and citation [6].

The USA, UK, Germany and other G7 countries are leading the world of research collaboration with increased international partnerships with each other and with other countries including those with low resources. The last decade has seen voluminous and qualitative growth in research globally with China and India leaping in bounds in local and international collaborative research, this particularly with US and UK. The increasing growth of scientific specialization should be harnessed to accommodate the needs of younger researchers in cross-fertilization of ideas and knowledge transfer across disciplines[2].

## International collaboration with low resource countries. Why?

Low resource countries are continuously being courted, particularly in health care research, as serious researchers from developed economies are seeking more collaboration with them because of the global nature of health problems, including environmental and genetic considerations [2]. The seeking out is bilateral, as researchers from low resource economies are also seeking collaboration particularly for the benefits of well equipped laboratory and intellectual facilities.

## Challenges of research collaboration with low resource countries

Major consideration to the low resource researchers is the question of why developed economies wish to collaborate with them. What are the goals of the particular research and its relevance to the health of the people in the low resource setting? Is it primarily in order to take something away in the giver-taker inequilibrum or as a development aid? At what cost or gain is it to the citizenry of the low resource country? These questions can be answered by proper ethical considerations, definition of long term effects and benefits with no harm to the low resource country [3].

In seeking collaboration with researchers in a low resource country, the principal investigator and others also need to be aware of the challenges facing the researcher and the research community in low resource settings. He must set relevant clear and transparent research goals and priorities in tune with opportunities available in the low resource country. As an example, a research topic such as “Prostate Cancer in the African Race” is rather fuzzy while “Prevalence of Microinvasive Prostate Cancer in Nigeria” is very clear and of relevance to Nigeria. Research ethics must be respected and socio-cultural considerations identified and adhered to as much as possible. Very often, there is need to aim at building in manpower training as well as improved research capacity into the research proposal. A definite plan must be made for a true partnership (not donor-recipient) and consideration for long term relationships planned. The dearth of proper training and well equipped laboratories in low resource countries is a source of discouragement for keen researchers in low resource countries, leading often to brain drain and poor output. More developed research centers could take this challenge by adopting low resource research centers and laboratories.

Researchers in low resource countries are not oblivious of examples of previous unacceptable collaborative processes, some of which had nothing to offer back but only came for and took data away [3]. Such researchers will naturally and in principle be averse to collaborations that do not play fair, hence it will be necessary for collaborators to make deliberate efforts to be seen as playing fair.

## Challenges facing the low resource country researcher

The challenges of research in low resource countries are multifaceted, including personal, institutional and national political factors and all these challenges directly affect collaborative efforts. National political instability caused by greed and power plays in low resource settings lead to impaired and incoherent planning, resource allocation and priorities. In such economies there may also be other overarching national health priorities like communicable diseases, education, malnutrition, illiteracy and poverty. For example in Nigeria, funding for research is difficult to decipher in the National Budget. Research funding is largely dependent on non-governmental organizations, most of which process also has to pass through some tortuous governmental bureaucracy. From personal experience, the researcher often has to put in up to 80-100% of funds personally.

Institutional Challenges in low resource economies include brain drain, lack of local funds, as well as a dearth of research laboratories and research centers [4]. Lack of basic infrastructure, e.g. water and electricity, inadequate and/or unavailable equipments may also constitute problems. Researcher challenges may also include lack of economic stability with associated poor pay and recognition leading often to being lured away from home countries and brain drain of institutions. There could be discouragement to do research due to lack of equipped training institutions, isolation, intellectual selfishness of other researchers and senior faculty.

## Overcoming the challenges

Overcoming national challenges facing the research atmosphere cannot be easy without major transparent and deliberate national policies favoring research capacity building [2]. National funding needs to be allocated and equitably distributed for setting up stand alone as well as institutional research laboratories. Non governmental bodies should be encouraged to fund research relating to their areas of service. Institutions need to deliberately prioritize the need to increase research activities. Such prioritizing will call for increased advocacy with politicians so that national challenges can be minimized. Institutions should increase local (intra and inter-institutional) research by encouraging the faculty with improved research laboratories and increasing the recruitment of junior research workers. International collaboration should be sought for greater capacity building. Researchers in low resource settings need to aim at getting trained, locally researching and publishing, as well as collaborating locally. They should seek scholarships, attend conferences, read widely and liberally search the internet for self improvement in areas of interest and beyond. They must be ready to invest personal time and money in search of personal capacity building [2,4].

## Summary

The gap in research capacity between low and high resource countries nor the global trend of health research cannot be ignored. Therefore the need for research collaboration between the two groups cannot be overemphasized. The discrepancy of economies makes for differences in research capacity, psychosocial needs, ethical considerations, focus and challenges. For such collaboration to work out, every collaborator needs to be aware of these challenges and work with them. Collaborations with low resource countries will require transparency, mutual respect and respect for social norms. Collaborations should no longer be that of taker or master, but of cooperation. The dearth of research manpower and well equipped laboratories in low resource countries, call for a deliberate effort by developed research centers to go a step further from collaboration and adopt a low resource research centre or laboratory.

## Competing interests

The authors declare that they have no competing interests.
